# Medicines Information and the Regulation of the Promotion of Pharmaceuticals

**DOI:** 10.1007/s11948-018-0041-5

**Published:** 2018-05-02

**Authors:** Teresa Leonardo Alves, Joel Lexchin, Barbara Mintzes

**Affiliations:** 1 Division of Pharmacoepidemiology and Clinical Pharmacology, Utrecht Institute for Pharmaceutical Sciences, WHO Collaborating Centre for Pharmaceutical Policy and Regulation, PO Box 80 082, 3508 TB Utrecht, The Netherlands; 2 Faculty of Health, York University, Toronto, ON Canada; 3 University Health Network, Toronto, ON Canada; 4 Faculty of Pharmacy and Charles Perkins Centre, The University of Sydney, Sydney, NSW Australia

**Keywords:** Pharmaceutical industry, Physicians, Promotion, Direct-to-consumer advertising, World Health Organization, Prescribing, Regulation

## Abstract

Many factors contribute to the inappropriate use of medicines, including not only a lack of information but also inaccurate and misleading promotional information. This review examines how the promotion of pharmaceuticals directly affects the prescribing and use of medicines. We define promotion broadly as all actions taken directly by pharmaceutical companies with the aim of enhancing product sales. We look in greater detail at promotion techniques aimed at prescribers, such as sales representatives, pharmaceutical advertisements in medical journals and use of key opinion leaders, along with the quality of information provided and the effects thereof. We also discuss promotion to the public, through direct-to-consumer advertising, and its effects. Finally, we consider initiatives to regulate promotion that come from industry, government and nongovernmental organizations.

## Introduction

Medicines can cure acute illnesses, treat chronic conditions, relieve symptoms and prevent future ill health. However, any decision to use a medicine involves weighing potential benefits against possible harms. To make an informed decision, a person needs information on the aims of the treatment, how it works, how to use it properly, the likelihood of benefit and harm, and how this medicine compares with other available treatment options or the option not to treat, as well as relative cost-effectiveness. The quality of information that accompanies medicines can make the difference between “a poison and a cure”, between a use that leads to better health and a use most likely to lead to harm. Of equal importance to information on medicines, inaccurate information on diseases and disease risks can lead to harm if patients seek medical treatment when it is not needed, leading to unnecessary medicine use and the potential exposure to drug-induced harm.

Irrational use of medicines is widespread. It includes the use when medicines are not needed, use beyond approved indications (off-label), choice of unnecessarily harmful or ineffective options, concomitant use of products that should not be combined, use by patients for whom there is no scientific evidence of benefit, excessive dosing, and the use of the more expensive of equivalent options. Underuse can also be a problem, for example if an inadequate dose or duration of use leads to treatment failure or development of resistance, or if a person fails to adhere or is not provided with the needed therapy, such as for example a patient with atrial fibrillation who does not receive an anticoagulant drug. Many factors contribute to inappropriate medicine use, including not only a lack of information but inaccurate and misleading promotional information.

The World Health Organization (WHO) defines the promotion of pharmaceuticals as, “*all informational and persuasive activities by manufacturers and distributors, the effect of which is to induce the prescription, supply, purchase and/or use of medicinal drugs*” (World Health Organization 1988). Not all promotion necessarily leads to inappropriate medicine use. However, a tension exists between the competitive pressures that manufacturers face to expand product sales, and support for limited, judicious use of the most cost-effective of available alternatives (World Health Organization Europe 1993). An analysis of the 25 most heavily promoted drugs in the United States (US) from August 2013 to December 2014 found that only one-third were rated as innovative, and only one was on the WHO’s essential medicines list (Greenway and Ross 2017).

Many forms of disguised promotion have flourished involving the use of scientific research and educational events to promote medicines’ sales (Fugh-Berman 2008). US court cases involving multinational pharmaceutical companies have uncovered a range of promotional activities raising strong ethical and public health concerns, such as the hiring of clinical expert ‘key opinion leaders’ to promote unapproved uses (off-label), ghostwriting of scientific articles, instructions to sales staff not to mention specific evidence of harm (Waxman 2005) and efforts to discredit clinicians who raised safety concerns (Rout 2009). Healthcare professionals frequently underestimate the extent to which their prescribing decisions are affected by the promotion of pharmaceuticals (Prosser et al. 2003) and most governments devote few resources to the regulation of promotion (Lexchin 2012).

The focus of this article is on the promotion of medicines to healthcare professionals and the public. We define promotion broadly as all actions taken directly by pharmaceutical companies with the aim of enhancing product sales. We highlight what is known about the quality of the information transmitted directly to doctors by sales representatives, journal advertising and key opinion leaders and then look at the major effects of these types of promotion on prescribing. We then specifically discuss promotion to the public through direct-to-consumer advertising and its effects. We chose these particular areas to focus on because there is concrete evidence that they affect prescribing patterns. The three authors have in-depth knowledge of the promotion of pharmaceuticals and have chosen representative evidence of key aspects of promotion, based on their expertise. Most, but not all, of the literature we cite comes from developed countries where the bulk of the research has been done. We conclude by reviewing initiatives from the pharmaceutical industry, government and nongovernmental organizations for improvement of the regulation of the promotion of pharmaceuticals.

## Spending on Drug Promotion

Global pharmaceutical sales reached $1057.1 billion in 2014 (Total unaudited and audited global pharmaceutical market 2005–2014 2015) (unless otherwise stated all monetary amounts are in US dollars). Medawar and Hardon describe a ‘crisis in innovation’ within the industry as a key driver of companies’ increased dependence on blockbuster drugs to maintain profitability, and hence of increased spending on promotion (Medawar and Hardon 2004). The marketing focus has recently switched to “niche-busters”, or high-priced drugs for smaller markets, but promotion is still needed to drive the sales of these products. Both total numbers of new molecular entities and the number with evidence of therapeutic advantage have declined in the last 50 years, without a decrease in industry profitability (Gagnon 2009). Compared with other industrial sectors, the pharmaceutical industry is the highest ranked investor in research and development (R&D) (Commission 2008). However, a report by the National Academy of Medicine puts marketing spending for the 12 largest pharmaceutical companies above $120 billion in 2016 compared to about $75 billion for R&D (National Academies of Science 2017).

Data on national spending on the promotion of pharmaceuticals are not publicly available in most countries; the US is a notable exception because of freedom of information laws and information that has become public in legal cases. Thus, much of the published research on promotional spending is US-based. Estimates of the amount spent in the US on promotion range from $57.5 billion in 2004 (Gagnon and Lexchin 2008) to $27.7 billion in 2010 (Kornfield et al. 2013). The 2004 figure was 24.4% of sales revenues, nearly double the amount spent on R&D and even the lower 2010 estimate represented 9.0% of sales. Most activities were directed at physicians and on average companies spent US$61,000 per physician in 2004. A 2014 audit by the market research company Cegedim confirms the primary focus on physician-directed promotion (Mack 2014b). Based on data released under the US Sunshine Act, in 2014 and 2015, companies provided US$2 billion in payments to individual US doctors per year, and another $600 million to teaching hospitals (Tigas and Ornstein 2016).

## Promotion of Medicines to Prescribers and Its Effects

### Sales Representatives

While the number of sales representatives of the products of the pharmaceutical companies in the US has dropped from a high of 105,000 in 2006 to 60,000 in 2013, the majority of doctors are still willing to see them (Mack 2014a). In 2015, just over a third of Canadian doctors did not see sales representatives, 11% saw 6 or more a month (Leslie 2015) and in that year there was a total of 3,720,000 visits (Canadian pharmaceutical industry review 2015 2016). Studies of oral presentations by sales representatives in Finland, Australia, the US, the Netherlands, and France, have found that information on harm is often omitted, and inaccurate information was consistently favorable towards the promoted product (Hemminki 1977, 1988; Prescrire Editorial Staff 2006; Roughead et al. 1998; Ziegler et al. 1995). More recently, a study of the frequency of safety information provision in 1692 promotions to family physicians in four cities in Canada, the US and France found that serious adverse events were rarely mentioned, even for products with US Food and Drug Administration (FDA) “black box warnings” of risks, and the minimum of information judged a priori to be needed for patient safety was provided only 2% of the time (Mintzes et al. 2013).

### Pharmaceutical Advertisements in Medical Journals

There are differences in national regulations concerning the information that must be provided in advertisements of pharmaceuticals, and differences in the extent to which national laws are enforced. However, as highlighted in WHO’s Ethical Criteria, specific elements of information should be included in order for advertisements to allow healthcare professionals to have a basic understanding of the promoted product (World Health Organization 1988). In practice, these elements are often missing even in advertisements in developed countries. A systematic review of the quality of pharmaceutical advertisements in medical journals identified 24 studies, reviewing advertisements from 26 countries, published between 1975 and 2006 (Othman et al. 2009). Although most of the advertisements provided the product’s brand and generic name, other information needed for rational prescribing, such as contraindications, interactions, side-effects, warnings and precautions were less commonly provided, and when supplied, were only available in the fine print. Interestingly, this information is required by the International Federation of Pharmaceutical and Manufacturers Associations (IFPMA) and European Federation of Pharmaceutical Industries and Associations (EFPIA) marketing codes (European Federation of Pharmaceutical Industries and Associations 2007; IFPMA 2007). Approved indications were stated in more than 70% of advertisements in five studies. However, a 2001 Russian study found that only 45% of advertisements mentioned approved indications (Vlassov et al. 2001). This was similar to results for the United Republic of Tanzania (40%) and Italy (34%) in an earlier multi-country study (Herxheimer et al. 1993). The systematic review of references referred to in advertisements found that few of them that provided supporting claims were methodologically rigorous and most had been funded by the manufacturer (Othman et al. 2009). Only 38% of the references were to clinical trials, systematic reviews or meta-analyses. References listed ‘data on file’ were often not supplied on request. The authors concluded that information quality is poor globally, with an impact expected to be greatest where access to high-quality independent pharmaceutical information is most limited. A comparative study of advertisements in medical journals in Australia, Malaysia and the US from 2004 to 2006 revealed that the majority of claims were vague and of poor quality, with fewer than one-third classified as unambiguous (Othman et al. 2010). This direct comparison found a problem of poor information quality in both wealthy industrialized settings and a middle-income country. By 2008 nearly half of physician-directed advertisements in US medical journals failed to adhere to at least one FDA guideline regulating content. In addition, advertisements did a poor job of conveying basic information necessary for safe prescribing, with most failing to quantify serious risks, more than one quarter failing to quantify benefits and nearly half providing no verifiable references (Korenstein et al. 2011).

Unsurprisingly, the situation in low and middle-income countries when it comes to journal advertisements is even more alarming than in high-income countries. A 2006 study in Bangladesh found that 34% of the claims in a sample of 116 brochures for family physicians were misleading (Islam and Farah 2007). In Zimbabwe, less than half of physician and pharmacy brochures for prescription drugs contained information on adverse effects, warnings and precautions, or major interactions (Sibanda et al. 2004). Similarly, in Nepal, promotional materials provided to hospital doctors in 2007 failed to mention adverse effects two-thirds of the time, and precautions, contraindications or warnings were only included in 36% (Alam et al. 2009). In Sri Lanka a considerable proportion of drug promotional materials collected in 2015 used poor quality scientific research as references (Kommalage et al. 2016). The majority of 200 pieces of drug promotional literature collected from departments in an Indian hospital in 2014 satisfied only half of the WHO criteria for rational drug promotion and none fulfilled all of the criteria (Ganashree et al. 2016). The situation in some developed countries was no better. In Germany in 2004, 94% of the claims in brochures directed at physicians failed to be supported by scientific evidence: in 15% no references were cited; in 22% the references could not be found; and in 63% the relevant study was cited but the claim differed from research results (Tuffs 2004).

### Key Opinion Leaders

One of the practices described by Steinman in 2006, in a report summarizing documents that became public in a legal case concerning off-label promotion, was the funding of clinician “key opinion leaders” (KOLs) to promote products (Steinman et al. 2006). Companies know that messages coming directly from them are likely to be viewed skeptically by physicians. As a result, the concept of using KOLs as an “independent” source of information has significantly expanded since the mid to late 1990s (Millard 2008). In the US, a 2007 survey found that 16% of physicians, or about 141,000 received payments for serving as a speaker or being part of a speakers’ bureau (Campbell et al. 2007). More recently, in just 5 months of 2013, companies made what appear to be speaker payments of $400 or greater to 55,000 doctors (Sismondo 2015).

Some KOLs are clinicians who are hired to give small-scale talks, but for major programs KOLs are typically well known and highly respected leaders in their field who are especially effective at transmitting messages to their peers. Pharmaceutical companies hire KOLs to consult for them, to give lectures, to run continuing medication education sessions, to conduct clinical trials, and occasionally to make presentations on their behalf at regulatory meetings or hearings (Elliott 2010).

Although most KOLs are “true believers” in the drugs that they are promoting they also readily acknowledge that there are other factors involved in their decision to work for drug companies, such as financial compensation, research funding, increase in the number of publications that they author, early knowledge about new drugs, being at the vanguard of their specialty and psychological rewards (Elliott 2010; Sah and Fugh-Berman 2013; Sismondo and Chloubova 2013).

One way of judging the importance that pharmaceutical companies place on KOLs is the case that roughly one-third of the marketing budget for pharmaceutical companies is spent on KOLs (Elliott 2010; Millard 2008). This amounts to an average of about $38 million on each product as it moves from clinical testing to launch (Pharma brands earmark $38 million for thought leaders 2006). Companies are willing to spend this amount of money because of the return that they get. According to an internal Merck document, doctors who attended a lecture by a KOL on Vioxx (rofecoxib) wrote an additional $623.55 worth of prescriptions for the drug over a 12-month period compared with doctors who didn’t attend. “After factoring in the extra cost of hiring a doctor to speak, Merck calculated that the ‘return on investment’ of the doctor-led discussion group was 3.66 times the investment, versus 1.96 times for a meeting with a sales representative” (Hensley and Martinez 2005). Whereas in 1998, in the United States, the number of talks by sales representatives and KOLs were about equal at just over 60,000 each annually, by 2004 there were almost twice as many talks by KOLs compared to sales representatives (Hensley and Martinez 2005)—a likely reflection of the economic benefits of using KOLs instead of sales representatives.

The talks that KOLs give can be scientifically valid but also deceptive, for example by touting the benefits of the company’s drug but not mentioning that other drugs are equally or more efficacious. Alternatively, KOLs may be hired to give presentations or write articles emphasizing the negative aspects of individual drugs or drug classes without ever mentioning the product made by the company paying them (Fugh-Berman 2005). Pharmaceutical companies need to maintain the fiction that KOLs are independent sources of information. This supposed independence is the main reason that doctors trust KOLs more than sales representatives. If KOLs are shown not to be independent then they lose their value to the companies. However, it is precisely when KOLs start to act independently and deviate from the messages that companies are cultivating, that their value to the company starts to be questioned (Norton 2000; Carlat 2007).

### Effects of Promotion to Prescribers

One of the drivers of inaccuracies and omissions in the promotion of pharmaceuticals is the need for each new brand to obtain and maintain market share, despite a frequent lack of scientific evidence of therapeutic advantage over existing treatment options. A French independent pharmaceutical bulletin, La revue Prescrire, evaluates the clinical trial evidence of effectiveness and safety for all new medicines and new indications for existing drugs approved in France. Only 1.3% of 1038 new drugs and/or new indications introduced between 2006 and 2015 were major therapeutic advances; 52% were “nothing new” and almost 17% should never have been marketed in the first place (Prescrire Editorial Staff 2016).

Although in some instances healthcare professionals admit that their prescribing could be influenced by seeing sales representatives (Workneh et al. 2016), in most cases they deny being affected. For example, in a survey of 446 physicians in Izmir, Turkey, Guldal and Semin (Guldal and Semin 2000) found that nearly two-thirds of the physicians thought that their prescribing was unaffected, although nearly half reported seeing sales representatives every day for 15 min or more. Belief in personal invulnerability does not necessarily extend to one’s colleagues: Steinman et al. found that 61% of US medical residents believed they were personally unaffected by promotion, but only 16% believed that their colleagues were similarly unaffected (Steinman et al. 2006). A study of medical students, interns and residents doing a psychiatry rotation found that the more gifts they reported receiving, the less likely they were to believe that they were affected (Hodges 1995). The feelings of reciprocal obligation brought about by smaller gifts should not be underestimated, as even tokens such as coffee mugs can have a surprisingly large effect (Katz et al. 2003). In a review of psychological and social science research on the effects of gift-giving, Katz and colleagues commented that, “Those who do not acknowledge the power of small gifts are the ones most likely to be influenced, because their defenses are down” (Katz et al. 2003).

A body of research evidence has shown a link between a greater reliance on the promotion of pharmaceuticals and less appropriate prescribing (Norris et al. 2005; Wazana 2000). A systematic review of empirical studies on the effects of promotion on physician behavior found that physicians with greater exposure had a higher prescription volume, prescribed more costly medicines, had more a rapid adoption of new medicines, including those without added therapeutic value, and made more requests for formulary inclusion of drugs without established therapeutic advantages (Wazana 2000). In 2010, a second systematic review examined the effects of exposure to information from pharmaceutical manufacturers on prescribing drug quality, volume and costs (Spurling et al. 2010). Among the 58 studies that met inclusion criteria, nearly all found either an association with lower quality, higher volume and higher costs, or no significant difference. The authors failed to find any evidence of net benefit from exposure to promotion. A more recent systematic review focusing solely on interactions between sales representatives and practicing physicians in developed countries echoed the findings of the study by Spurling et al. Fifteen out of the 19 included papers found a consistent association between interactions promoting a medication, and an inappropriate increase in prescribing rates, lower appropriateness of the prescribing, and/or increased prescribing costs (Brax et al. 2017).

More focused research has reinforced the general findings from these systematic reviews. Muijrers et al. evaluated prescribing quality using 20 indicators based on Dutch general-practice guidelines, combining physician survey data and administrative records (Muijrers et al. 2005). General practitioners (GPs) who saw sales representatives more often were significantly less likely to adhere to these guidelines. A double-blind randomized controlled trial in the US tested the effect on conscious and unconscious attitudes of exposing medical students to small branded promotional items (Grande et al. 2009). Students’ attitudes were affected by the institutional policies of their medical faculties. One of the medical faculties involved in the study had restrictive policies towards the pharmaceutical industry, while the other was more permissive. In the less restrictive environment, small gifts created a more favorable attitude to the brand; in the more restrictive environment, students receiving gifts had less favorable attitudes. Prosser et al. examined the influences on 107 UK GPs’ initial prescriptions for 15 new drugs (Prosser et al. 2003). Physicians cited sales representatives as influential 39% of the time, more often than all other influences, including patient-related factors such as suboptimal current therapy. These results reinforce the influence that sales representatives have, even if this influence is rarely cited by doctors when asked a general question on prescribing. Studies in the US and France similarly found that sales visits were predictive of initiation of psychiatric treatment in primary care (Schwartz et al. 2001; Verdoux et al. 2005), and in Denmark, sales visits were associated with a shift in the brand of inhaled corticosteroid prescribed for asthma, but not overall prescribing volume (Sondergaard et al. 2009).

## Promotion of Prescription Medicines to the Public and Its Effects

The prohibition of direct-to consumer advertising (DTCA) in most countries is a health protection measure linked to prescription-only status. Prescription-only medicines generally treat more serious conditions that cannot be easily self-diagnosed and are generally more toxic or have a less well-understood toxicity profile than non-prescription medicines.

DTCA of prescription medicines has grown rapidly in the US and New Zealand, the only two countries where it is legal. In late 1997, the FDA introduced an administrative policy allowing companies to list only major and frequent risks in broadcast advertising as long as sources of more complete information were provided. This shift opened up television to DTCA. By 2000, advertising spending for top ‘blockbuster’ medicines had surpassed brands such as Pepsi Cola, Budweiser beer or Nike shoes (Findlay 2000). Spending in this area in the US grew by 19% from 2014 to 2015 to a total of $5.4 billion (Bulk 2016).

One of the top five brands, in terms of spending in the early 2000s was rofecoxib (Vioxx), an arthritis medicine that was later withdrawn in 2004 due to cardiac risks. This was not the first advertised medicine to be withdrawn from the US market since DTCA became widespread. However, because of extensive potential harm from widespread rofecoxib use (Graham et al. 2005), some of which was stimulated by DTCA, US DTCA policy began to be scrutinized. Despite congressional hearings, new industry self-regulatory guidelines, and legislative proposals to restrict DTCA, in the 8 years since the withdrawal of rofecoxib, the practice has continued largely unchanged (Mintzes 2012). The FDA directly regulates advertising content, and regularly finds advertisements to be illegal, generally because of inadequate risk information or exaggeration of benefits. An analysis of 10 years’ worth of magazine advertisements indicates that most failed to include basic information needed for shared and informed treatment choices (Bell et al. 1999). An analysis of DTCA that appeared on television between 2004 and 2007, found frequent use of emotive imagery linking medicine use with happiness and social approval (Frosch et al. 2007), and a review of advertisements airing between 2008 and 2010 concluded that 55% of claims made for the drugs being promoted were potentially misleading (Faerber and Kreling 2013). However, according to a 2006 report by the US Government Accountability Office (GAO), the agency was able only to monitor a small—and shrinking—proportion of advertisements, due to the rapid growth in volume (United States Government Accountability Office 2006) and there is no indication that the situation has changed in the decade since the GAO issued its report. New Zealand relies on industry self-regulation, with all advertisements subject to pre-screening, but information criteria are weaker than those applied by the FDA, with little risk information required in advertisements (Hoek 2002; Every-Palmer et al. 2014).

Proponents of DTCA claim that it helps empower consumers and stimulates discussions with physicians, that it enables patients to obtain needed treatment at an earlier stage and improves adherence. There is no reliable evidence to back claims that DTCA leads to improved access to needed medicines, adherence, or patient and consumer autonomy (Frosch et al. 2010). Manufacturers have also begun to run compliance programmes for patients who are taking a specific brand. This has led to concerns that patient safety may be compromised, particularly if DTCA has led to patients taking a medicine with a poor safety profile or inadequate efficacy (Prescrire Editorial Staff 2007).

There is evidence that the rates of diagnoses of specific conditions increases during associated advertising campaigns, but the extent to which this is needed treatment or increased use among those unlikely to benefit remains an open question. If the threshold for diagnosis of a health condition shifts to include milder health problems, increased rates of diagnosis and treatment do not necessarily lead to health benefits. Kravitz and colleagues carried out a randomized controlled trial of the influence of patient requests for paroxetine (Paxil) using standardized patients who described symptoms of depression or of a less severe temporary condition, “adjustment disorder” that is due to life problems and does not require antidepressant treatment (Kravitz et al. 2005). If patients requested an advertised brand, they were equally likely to receive an antidepressant prescription whether they had symptoms of depression or “adjustment disorder”. A pharmaco-economic model of the effects of antidepressant advertising found a net benefit, although only six of every 100 people treated with antidepressants were expected to have depression (Block 2007). However, this model omitted any adverse effects, included unrealistic assumptions of the magnitude of treatment success, and underestimated associated health-care costs. When these were included in a similar model, harm largely outweighed benefit (Jureidini et al. 2008). DTCA for testosterone therapies in the US between 2009 and 2013 was associated with increased testosterone testing and more initiation of testosterone use without recent serum testing, contrary to treatment guidelines (Layton et al. 2017).

In Turkey, the government suspended the market licence for bupropion (Zyban) for 3 months in response to an illegal DTCA campaign (Semin et al. 2007). Turkey represents an example of a middle-income country that has experienced rapid growth in pharmaceutical spending. In 1998, 35% of total health-care spending was on pharmaceuticals, a greater relative proportion than in higher-income countries (Savas et al. 2002). Prescribing rates are higher than in most industrialized countries, polypharmacy is a serious problem and enforcement of prescription-only status poor. Semin and colleagues discuss the extra vulnerability of countries like Turkey to harm from DTCA both through increased spending in this area and because prescription-only status is poorly enforced, so a person may buy an advertised medicine without visiting a doctor (Semin et al. 2007).

Canada has been subject to strong pressure to introduce DTCA (Merck Frosst June 17, 1996; Mintzes 2006) and US magazines, cable and satellite television reach Canadian audiences with prescription drug advertising that is illegal under Canadian law (Mintzes 2008). Researchers compared prescribing rates for three drugs advertised in US media in English-speaking Canada versus Quebec, which is mainly francophone, and thus is less affected by US media. They found an effect on prescribing rates for one product, tegaserod (Zelnorm), a drug that was since withdrawn from the market for safety reasons (Law and Soumerai 2008). No effect on prescribing was seen for the other two drugs: mometasone, which was reimbursed without restrictions in Quebec, but not in most English provinces, and etanercept (Enbrel), a medicine provided in specialist care to a limited group of patients. These findings strongly suggest that the success of DTCA in stimulating sales is mediated by other factors such as reimbursement status (Hansen et al. 2005).

There are many forms of advertising of medicines on the Internet, including sites that directly offer products for sale, with few controls to ensure the quality, efficacy and safety of promoted products, or to ensure the accuracy of promotional claims. A study commissioned in 2009 by the Netherlands Health Inspectorate analyzed 41 web sites offering health information in the Dutch language: 32 were either hosted or sponsored by a pharmaceutical company, and 23 (72%) contravened national regulations by referring directly or indirectly to a specific prescription medicine (Nuland et al. 2009). An analysis of the top 50 web sites on schizophrenia on Google and Yahoo (n = 64 in total) identified 58% as pharmaceutical-industry funded (Read 2008). Those funded by industry were significantly more likely to support biological or genetic causes and to ignore the role of psychosocial stressors, to emphasize medication rather than psychological treatments, to portray schizophrenia as a more debilitating and chronic illness, and to discuss risks of violence if patients came off medication. John Read, the author of this study, comments: “For the websites examined in this study, perspectives and statements that are likely to increase drug sales are significantly more likely to appear on web sites funded by drug companies.” In its warning letters and notices of violations from 2005 through 2014 related to online promotional activities of prescription drugs advertised directly to consumers, the FDA expressed considerable concern over this type of promotion for cancer treatments, which require regular prescriptions for long-term treatment. The most significant finding from an analysis of these documents suggests that the online promotional content of prescription drugs fails to present risks and benefits in a balanced manner (Kim 2015).

Pharmaceutical companies also target the public directly through disease awareness campaigns or help-seeking advertisements. These materials usually contain health and disease information which focus on symptoms and suggest a visit to the doctor to learn more about new treatments. A study explored examples of such campaigns in printed media in the Netherlands by assessing their compliance with the WHO Ethical Criteria for medicinal drug promotion and the Dutch guidelines for provision of information by pharmaceutical companies. It concluded that although brand names were not mentioned in the campaigns, there was an overwhelming lack of compliance with the regulations mainly due to a lack of balance, absence of a listed sponsor, use of misleading or incomplete information and use of promotional information (Leonardo Alves et al. 2014).

## Initiatives to Regulate Promotion

### Pharmaceutical Industry

Most major pharmaceutical companies have their own codes of ethics that, in some countries, involve disclosure of information on payments to third parties. In the US, Eli Lilly discloses both individual and organizational funding on its website (www.lilly.com), and Pfizer discloses funding to organizations (www.pfizer.com) (Steinbrook 2007). Rothman and colleagues examined the profile of ‘health advocacy organizations’ funded by Eli Lilly, including both patient and professional groups (Rothman et al. 2011). The conditions these groups represented were highly correlated with Eli Lilly drug sales; in the case of both groups, neurosciences, endocrinology and oncology conditions predominated. Although the company had disclosed financing, only 25% of funded organizations did so, and only 10% acknowledged Lilly funding of events. This study highlighted the importance of manufacturer disclosure of funding, and the need for concurrent stronger standards for transparency among funded organizations.

National industry organizations in nearly all developed countries and many developing countries also have codes of marketing practice or ethics (Francer et al. 2014). The 2009 version of the US PhRMA Code of Ethics, still used at present, bans many traditional forms of gifts, such as pens, mugs and other office items. However, it still allows certain types of gifts if considered to be “items designed primarily for the education of patients or healthcare professionals” valued at $100 or less. The Code also allows the provision of meals and free samples (Pharmaceutical Research and Manufacturers of America 2008). Importantly, this is a voluntary self-regulatory code with no provisions for sanctions. Australia relies primarily on industry self-regulation of promotional practices, but ultimately the national government is responsible for upholding the law. In 2006, the Australian Competition and Consumer Commission required changes to the industry’s voluntary code of marketing, requiring companies to report details of funding for educational events, including venue, purpose, amount of hospitality provided, number of attendees and total cost. The data must be provided to Medicines Australia, the industry association, every 6 months, and Medicines Australia is committed to making the information publicly available as well as investigating any potential promotional violations (Australian Competition and Consumer Commission 2006). Medicines Australia has also instituted substantial fines for promotions that breach its Code of Conduct (Medicines Australia code of conduct breaches 2008). Jane Robertson and colleagues reviewed the experience with mandatory disclosure from July to December 2007, the first period for which the policy was in effect (Robertson et al. 2009). There were nearly 15,000 sponsored events over this period, or around 600 per week, at a cost of approximately $880 thousand per week. The company had some influence on educational content 91% of the time, and two-thirds of the events were for medical specialists. Robertson and colleagues argued that although this is an important shift towards greater transparency—a direction that many more self-regulatory agencies should adopt—more comprehensive reporting is needed. For example, details such as the names of speakers at educational events, whether sponsors selected speakers or influenced the content of the event, and whether there are direct or indirect financial ties between the speakers and the sponsors could allow better assessment of the educational value of the sponsored events.

The next analysis of industry sponsored educational events in Australia was published 10 years later, in 2017 (Fabbri et al. 2017). This long gap likely occurred because company reports were posted on the Medicines Australia website as separate pdfs, and not in an analyzable format. Over a four-year period, from October 2011 to September 2015, pharmaceutical companies hosted over 116,000 events for healthcare professionals in Australia, at a total cost of over AU$286 million. Most events were held in a clinical setting and in nearly all, sponsors provided free food and drink. There were on average just over 600 events per week, as in 2007. This study confirms just how widespread the “drug lunch” is at a national level. From 2015 on, Medicines Australia removed reporting requirements for food and drink and capped payments at AU$120 per person. Based on the 2011–2015 data, this would mean that two-thirds of events would no longer be reported, as food and drink were the sole expense (Fabbri et al. 2017).

Two of the strongest European self-regulatory codes are reputed to come from industry associations in the United Kingdom and Sweden. An analysis of antidepressant advertisements in Swedish medical journals between 1994 and 2003, concluded that companies failed to provide reliable antidepressant information and that this failure may be attributable to lax oversight, combined with the lag between when an advertisement was printed and when the company was censured and low fines for violations (Zetterqvist and Mulinari 2013). The ability of the self-regulatory codes in both countries to adequately monitor and control promotion was further called into question in an examination of code complaints, complainants and rulings for the period 2004–2012. Fines for code violations averaged in total €447,000 and €765,000 per year in Sweden and the UK, respectively, equivalent to about 0.014 and 0.0051% of annual sales revenues of pharmaceuticals, respectively. According to the authors, the prevalence and severity of breaches testifies to a discrepancy between the ethical standards codified in industry codes and the actual conduct of the industry (Zetterqvist et al. 2015).

In Canada, a multi-stakeholder organization that includes not only the industry but also representatives of professional groups, consumers, the media and the advertising industry, the Pharmaceutical Advertising Advisory Board (PAAB), prescreens all journal advertisements and advertisements in other media including print, audio, visual, audio/visual, and on-line advertising (Pharmaceutical Adverising Advisory Board 2013). Although the PAAB operates on a voluntary basis all the companies that belong to the research-based pharmaceutical industry association in Canada have agreed to abide by its code. There are some strong features of the code such as the requirement that if relative risk reductions are used to present benefits in advertisements the absolute risk reduction or number needed to treat also needs to be given or there needs to be information in the advertisement to calculate these values. However, the PAAB also has significant weaknesses; 5 out of the 13 members on its board come from associations that directly benefit from pharmaceutical advertising and most others receive pharmaceutical industry funding. Additionally, similar to the PhRMA code, there are no significant sanctions for violating the PAAB code.

In the European Union, the European Federation of Pharmaceutical Industries and Associations (EFPIA) adopted a Code of Practice on Relationships between the Pharmaceutical Industry and Patients Organisations in 2007 (European Federation of Pharmaceutical Industries and Associations 2007), which was later amended in 2011 and again at the end of 2013. Under this self-regulatory code, all EFPIA member companies are requested to publish the names of the patient organizations they support. EFPIA hosts a list of their 31 members that voluntarily declare patient group sponsorship and provides links to their web sites (European Federation of Pharmaceutical Industries and Associations 2011). An investigation by Consumers International (CI) of the actual promotional practices by 20 large multinationals operating in the European Union in the mid 2000s found that only two, GlaxoSmithKline and Novartis, reported the number of confirmed marketing code breaches and resulting sanctions. CI could not find any information about the European marketing policies for 8 companies. According to CI “[t]he absence of clear marketing policies for these companies is remarkable, given that irresponsible marketing practices form a serious, persistent and widespread problem among the entire pharmaceutical industry…A particularly worrying trend shown by our research is that the difference between policies and practices is often striking” (Consumers International 2006). At the time of writing of this article (February 2018) there is no evidence of any subsequent investigation to ascertain whether the amended EFPIA code has altered the situation.

EFPIA brought in a transparency code in 2013, requiring national member industry associations to implement policies on disclosure of payments to healthcare professionals if they were not already subject to legal requirements to do so (Santos 2017). However, these policies suffer from several drawbacks such as incomplete reporting because of exclusion of certain payments such as food and drinks, individual healthcare professional consent that amounts to an “opt-out” clause, and in some cases lack of centralized reporting or posting within a searchable database.

The IFPMA updated its 2000 self-regulatory code of marketing practices in 2006, released in January 2007, and later further updated in 2012 (IFPMA 2007, 2012). The IFPMA Code is the only regulatory standard in countries without government regulation of drug promotion or a national industry association with a self-regulatory code. The 2006 and 2012 versions of the Code are longer than the version they replaced and include more explanatory sections. However, they fail to explicitly cover two promotional activities that were covered in the 2000 code: the activities of pharmaceutical sales representatives, and direct-to-consumer advertising of prescription drugs (Putzeist 2007). Neither omission is likely to be an oversight as these are key promotional activities. The 2006 and 2012 versions of the code also lack any provisions for the active monitoring of promotional activities.

### Government Regulation

When a medicine is approved for marketing, it includes the pharmaceutical product and accompanying packaging, information, labelling and package inserts. Approved product information is prepared by the manufacturers and summarizes the scientific evidence on effects and sets out conditions for use, although it is subject to approval by regulatory agencies. It also sets out a basis for the judgment of which promotional practices are and are not permitted, and criteria they must meet to comply with national law, such as requirements for consistency with approved indications and product information or prohibitions of financial incentives that may influence prescribing.

Governments may take a more or less direct role in the regulation of the promotion of pharmaceuticals. For example, in some countries, the regulatory agency is directly responsible for pre-approval, monitoring, response to complaints, and levying of any fines and other sanctions. In other cases, most of these activities are delegated to an industry self-regulatory body or a multi-stakeholder committee that is independent of government. Others may opt for a co-regulatory approach, in which the government agency has responsibility over certain forms of promotion, the industry self-regulatory body over others. In general, few resources are devoted to the regulation of the promotion of pharmaceuticals in either low- or higher-income countries (Buckley 2004; Lexchin 2012), despite the evidence that promotion strongly affects prescribing and medicine use, and the public health implications and costs to society of these effects (Spurling et al. 2010).

The WHO Ethical Criteria for Medicinal Drug Promotion, published in 1988, remain the global standard for the promotion of pharmaceuticals with an explicit aim to support the rational use of medicines. Although new media and marketing techniques have emerged in the 25 years since their development, key criteria still cover the major promotional issues of concern. For example, one such principle is the avoidance of “…misleading or unverifiable statements or omissions likely to induce medically unjustifiable drug use or to give rise to undue risks”.

Since 1999, the WHO has carried out surveys of all United Nations (UN) Member States every 4 years on the ‘structures and process of country pharmaceutical situations’, including the regulation of the promotion of pharmaceuticals. Countries are divided into low, middle and high income according to their per capita gross national product. In 2003, 148 (77%) of UN Member States responded, in 2007, 150 (78%) (World Health Organization 2007). Table 1 presents the key results concerning regulation of drug promotion for these two surveys. The results are not directly comparable, as there were differences in how the questions were posed, and the 2007 survey elicited more detailed information. Most national governments reported that they had national medicines legislation and legislation specifically covering the promotion of pharmaceuticals. Law enforcement varied by income level, with only one-third of high-income countries reporting that they relied solely on government regulation, as compared with most low-and middle-income governments. Few national governments reported reliance solely on industry self-regulation. In 2007, nearly 2/3 of high-income country governments reported reliance on co-regulation. The rate was lower in 2003, but there were also fewer survey respondents from high income countries. In most countries that adopt a co-regulatory approach, enforcement of the law is primarily delegated to an industry self-regulatory body. For example, in the UK, the industry association, the Association of the British Pharmaceutical Industry, has a code of practice that is enforced through the Prescription Medicines Code of Practice Authority (see: www.pmcpa.org.uk/). The national regulatory authority is able to step in, should self-regulatory approaches fail or intervention be deemed necessary, for example if an imminent risk exists to public health. In practice, such interventions are infrequent.

**Table 1:** Regulation of pharmaceutical promotion: World Health Organization survey results 2003 and 2007 (all percentages are of column totals)

	Low income (per capita income ≤ US$935/year)		Middle income (per capita income US$936-11,455/year)		High income (per capita income ≥ US$11,456/year)	
Number of respondents^a^	2003 (n = 57)	2007 (n = 47)	2003 (n = 65)	2007 (n = 69)	2003 (n = 18)	2007 (n = 34)
National medicines legislation	52 (91%)	41 (87%)	55 (85%)	60 (70%)	18 (100%)	32 (94%)
Law on promotion	47 (82%)	41 (87%)	50 (77%)	59 (86%)	16 (89%)	34 (100%)
Promotion regulated by						
Government	38 (67%)	40 (85%)	32 (49%)	48 (70%)	6 (33%)	12 (35%)
Industry self-regulation alone	2 (4%)	2 (4%)	1 (2%)	3 (4%)	1 (6%)	0
Co-regulation^b^	6 (11%)	2 (4%)	14 (22%)	11 (16%)	8 (44%)	22 (65%)
NGOs/civil society involved^c^	8 (14%)	6 (13%)	15 (23%)	20 (21%)	8 (44%)	11 (32%)
Types of regulation (2007 data only)	Low income (n = 47)		Middle income (n = 69)		High income (n = 34)	
Advertising pre-approved		26 (55%)		40 (58%)		11 (32%)
DTCA^d^ of prescription medicines banned		36 (77%)		43 (62%)		21 (62%)
OTC^e^ ads regulated		20 (43%)		31 (45%)		15 (44%)

^a^ In 2003, national income level data were available for 140/148 (95%) countries who responded (148/191 (77%) of UN Member States); in 2007 income was available for all 150 respondents (150/192 (78%) of UN Member States). Listed income cut-offs are 2007 criteria

^b^ Co-regulation refers to joint government and industry regulation. In 2003, no direct questions were posed on co-regulation. Countries that reported they relied both on government regulation and industry self-regulation are listed as having a co-regulatory approach

^c^*NGO* nongovernmental organization

^d^*DTCA* direct-to-consumer advertising of prescription medicines

^e^*OTC* over-the-counter medicines

There has been little evaluation of the effectiveness of varying approaches to the regulation of the promotion of pharmaceuticals. If there are inadequate resources for monitoring, government involvement in regulatory activities may be minimal to non-existent, both in higher- and lower-income countries. For example, a 2003–2004 parliamentary investigation in Canada noted that no fines had been allocated for any promotional regulatory violations during the past 25 years (House of Commons Standing Committee on Health 2004). In contrast, from 2004 to 2007 Brazil levied fines totaling around $10 million for 959 infringements of the law on pharmaceutical advertising (Rocci 2009). The US has the strongest history of fining pharmaceutical companies; since 1991, they have paid $35.7 billion in civil and criminal penalties (Almashat et al. 2016). However, profits generated through violations far outweigh even these fines (Evans 2009).

In 2002, WHO published a 10-country comparison of the regulation of medicines, including the regulation of the promotion of medicines (Ratanawijitrasin and Wondemagegnehu 2002). The sample included all six WHO regions and a variety of national income levels. In one of the 10 countries, Cuba, no pharmaceutical advertising or promotion was allowed. In the other nine, the authors noted that, “The empirical data for assessing the regulation of drug information [promotion] are highly inadequate. Even records of the number of violations and the percentage of each type of sanction imposed are generally unavailable. So, too, is information on the effectiveness of action to prevent inaccurate and misleading drug information from reaching health care providers and the public.”

A 2007 study analyzed the regulation of advertising and promotion of pharmaceuticals in seven countries in Latin America, in relation to the WHO Ethical Criteria. Relevant legislative texts and regulations were collected in Argentina, Bolivia, Brazil, Colombia, Ecuador, Nicaragua and Peru. The aim was to examine the consistency of national approaches to regulation with the WHO Ethical Criteria and to evaluate their content, restrictions and flexibilities. The study concluded that while the Ethical Criteria acted as a reference in the setting of norms, there was a tendency to exclude key concepts necessary to prevent harm and protect health. Ample room was provided for interpretation and there was recurrent use of vague wording, for instance, in the definitions of promotion, advertising and medical information. The latter enabled the dissemination of disguised promotion to the public. In addition, there was little information on enforcement and sanctions or the role to be played by consumers and independent organizations in the monitoring of the promotion of pharmaceuticals (Vacca et al. 2009).

Some governments have introduced improvements in regulation. Initially in 2000 and later in December 2008, Brazil introduced broad changes to the regulation of the promotion of pharmaceuticals aiming to extend the scope of existing regulations. An analysis of over 800 advertisements in Curitiba (the capital of Paraná State), published in 2007, thus predating the 2008 regulatory changes, had found that three quarters failed to comply with regulations and on average there were 4.6 infractions per advertisement (Wzorek et al. 2007).The changes included additional controls on advertising of over-the-counter medicines, such as prohibition of celebrity endorsements and product placement in television, radio, films or theatre productions. The active ingredients must be stated and advertisements must include warnings, such as contraindications for use in young children or during pregnancy. These legal changes also introduced limits on the volume of free samples and prohibition of gifts for physicians or pharmacists (Bruce 2008).

In 2010, the Physician Payments Sunshine Act, part of US health reform legislation under the Affordable Care Act, enacted provisions requiring all pharmaceutical industry payments above $10 to physicians to be publicly disclosed. Starting in September 2014 these payments were available through the Open Payments website of the Centers for Medicare and Medicaid Services (Lenzer 2016). Some major medical faculties in the US, including Stanford University, have gone further than national governments or industry self-regulatory bodies to implement both full disclosure and limits on the types of industry financing faculty members may accept. For example, participation in industry speakers’ bureaus is not permitted (Stanford University 2006). The US legislation has allowed researchers to examine the effects of payments to doctors on their prescribing behavior. One study linked the Sunshine Act data for 2013 with prescribing information obtained from the Medicare Part D database, the US federal program that covers prescriptions for the elderly. The authors found that the receipt of payments was associated with greater prescribing costs per patient, and more prescribing of branded medicines (Perlis and Perlis 2016). A second study using the same datasets showed that the receipt of industry-sponsored meals of even $20 or less was associated with increased prescribing of the brand-name medication that was being promoted at the meal (DeJong et al. 2016).

In France, broad regulatory changes were brought in following the Mediator (benfluorex) scandal, a medicine withdrawn for safety reasons in 2009. benfluorex was approved for use in type 2 diabetes, but was widely prescribed for weight loss, an unapproved use. Benfluorex causes heart valve abnormalities, similar to two closely related medicines (fenfluramine and phentermine) that had been withdrawn from the market globally 12 years earlier. L’Inspection générale des affaires sociales, a French regulatory authority, investigated the factors contributing to this long delay in taking action on safety concerns and found that conflicts of interest in regulatory decision-making had played a large part (Morelle et al. 2011).

In Portugal, legislation passed in 2013 requires disclosure by healthcare professionals (individuals or associations), hospitals and other health institutions as well as patient organizations of any subsidy, sponsorship or gift received from the pharmaceutical or medical device industries. Similarly, sponsors are also required to declare any support they provide on the online portal hosted by the Portuguese drug regulatory agency (Comunicações ao INFARMED, I.P., no âmbito da Transparência e Publicidade de acordo com o Artigo 159.º do Decreto-Lei n.º 176/2006, de 30/08 (Medicamentos) e, com o artigo 52.º do Decreto-Lei n.º 145/2009, de 17/06 (Dispositivos Médicos) 2009). Other European countries with legislation requiring disclosure of industry payments to healthcare professionals include France, Greece, Romania and Latvia (Santos 2017).

### Recent Nongovernmental Initiatives

As is noted above, academic clinicians have a major role in some of the newer ‘non-traditional’ forms of pharmaceutical promotion blurring distinctions between science or education and advertising. The financial ties between clinical experts and pharmaceutical manufacturers are often unclear. This has implications not only for clinical practice, but for the roles of researchers and educators within medicine and the other healthcare professions.

Since March 2007, France’s public health code has required healthcare professionals to declare their financial links to pharmaceutical manufacturers in any relevant public statements in print or broadcast media (République Française 2002). Despite this requirement, a physicians’ organization, Formindep (pour une Formation et une information médicales indépendantes), together with the consumer group Que choisir have filed charges against nine KOLs who are considered leading experts in their field for failing to declare their ties to manufacturers when speaking publicly (Lenzer 2016).

In the US, there have been some initiatives to address the influence of pharmaceutical funding of academic physicians. One driving force behind these institutional changes has been the American Medical Students Association (AMSA) PharmFree Scorecard initiative. Since 2007, AMSA has published grades for all medical faculties within the US on conflict of interest policies involving faculty, the presence of sales representatives and free samples in teaching hospitals, and industry financing of educational activities (American Medical Students Association 2009). Institutional policies are published on the web in a format that facilitates comparisons and provides full descriptions both of exemplary policies and those judged to be inadequate.

Acción Internacional para la Salud Nicaragua has developed a short module on critical appraisal of the promotion of pharmaceuticals, to be incorporated into workshops and other educational events outside of the formal curriculum. Together with the Drug Research Utilization Group of Latin America (www.durg-la.uab.es), a working group was created to implement this module in universities across Latin America. Institutions in Argentina and Colombia adapted it and implemented the module as part of their curriculum. The module package includes reference materials (national legislation, WHO Ethical Criteria), tools for critical appraisal (examples of advertisements, frameworks for analysis, independent information and videos), as well as an evaluation tool to measure its impact. Between 2006 and 2009, 1346 medical students and 200 pharmacy students attended the module at five universities in Nicaragua, Argentina and Colombia. A large majority of participants considered the module to be useful and relevant to their education as healthcare professionals and would recommend it to their colleagues. In Nicaragua, the evaluation revealed that the module raised awareness about the interactions between health staff and the pharmaceutical industry but also improved critical appraisal skills during sales representative visits and in the analysis of printed advertising materials (Vacca 2010).

Another initiative has focused on curriculum development and testing to improve training of medical and pharmacy students about the promotion of pharmaceuticals and the ethical choices they will face once in practice concerning relations with industry. A manual was produced in 2009 in English, Spanish and Russian, and in 2013 in French: “Understanding and Responding to Pharmaceutical Promotion, A Practical Guide” (Mintzes et al. 2010). This curriculum development is a joint project of WHO and Health Action International, an international NGO. A revised version of this manual was developed specifically for healthcare professional students in the European Union (Fact or fiction? What healthcare professionals need to know about pharmaceutical marketing in the European Union 2016).

## Conclusion

In conclusion, the WHO Ethical Criteria for Medicinal Drug Promotion remain a global gold standard for the regulation of drug promotion, on which national regulations and codes can be based. Unfortunately, the implementation of the WHO Ethical Criteria remains incomplete, and many widespread new forms of drug promotion are in clear violation of the criteria. These include, for example, the use of clinician key opinion leaders, continuing medical education and disease mongering, as vehicles for disguised promotion.

There are some positive trends in the regulation of the promotion of pharmaceuticals, such as rules requiring mandatory disclosure of funding of healthcare professionals and patient groups, but more systemic fundamental changes are still needed. To realize the full potential health benefits of medicines as a social good, expanded professional and public access to accurate information from the industry, as well as independent, comparative information, is needed, with a clearer distinction between commercial activities and health-care provision and use. The industry has a role in ensuring that approved product information is widely available, and that the full protocols and reports of results of all sponsored clinical trials are made public, as well as post-marketing safety and effectiveness information.

There is ample evidence that promotion affects patterns of prescribing and medicine use, with effects on costs and on appropriateness of medicine use. Regulation aims to ensure that promotional messages are consistent with the scientific evidence and public health objectives, but has been under-resourced, with little to no evaluation of ‘best practices’ in regulation of promotion, or what does and does not work.

In order to ensure that the needs of patients and the public—the users of medicines—are at the center of medicine use decisions, both better access to high quality independent information and stringent regulation of drug promotion are needed. This can only be accomplished if the political will exists to ensure that national governments give priority to the health needs of citizens over the need for national and international industries to expand their markets. Where self-regulatory bodies exist, they should function in an open and transparent manner, with full publication of complaints and decisions, and include firewalls between member companies and the committees that judge whether or not promotional practices violate industry norms.

For national governments aiming to better manage medicine use so as to maximize health benefits and cost-effectiveness, two complementary approaches to promotion of rational medicine use are needed, ideally situated within a broader national medicines policy. First, improvements in the regulation of the promotion of pharmaceuticals are a necessary precondition to promotion of rational medicine use. These must address both direct and disguised or indirect forms of the promotion of pharmaceuticals, including the use of expert clinicians as key opinion leaders. Second, there is also a need for publicly-financed, independent/non-commercial information to be integrated into health service provision. Even the best-regulated promotion of pharmaceuticals by definition aims to sell a product, and cannot replace independent/non-commercial, unbiased comparative information sources.
